# Establish a Novel Model for Predicting the Risk of Colorectal Ademomatous Polyps: a Prospective Cohort Study

**DOI:** 10.7150/jca.74772

**Published:** 2022-08-15

**Authors:** Wenjie Li, Zhe Chen, Han Chen, Xu Han, Guoxin Zhang, Xiaoying Zhou

**Affiliations:** Department of Gastroenterology, The First Affiliated Hospital of Nanjing Medical University, Nanjing, Jiangsu, China.

**Keywords:** Colorectal ademomatous polyps, Colorectal cancer, Prediction model, Occurrence risk

## Abstract

**Purpose:** To establish and validate a model to determine the occurrence risk of colorectal ademomatous polyps.

**Methods:** A large cohort of 3576 eligible participants who were treated in the Department of Gastroenterology, the First Affiliated Hospital of Nanjing Medical University from June 2019 to December 2021, were enrolled in our study and divided into discovery and validation cohorts at a ratio of 7:3. LASSO regression method was applied for data dimensionality reduction and feature selection. The nomogram for the occurrence risk of colorectal ademomatous polyps was constructed based on multivariate logistic regression. The predictive performance of the model was evaluated regarding its discrimination, calibration, and clinical applicability.

**Results:** A total of 10 high-risk factors were independent predictors of the colorectal ademomatous polyps occurrence and incorporated into the nomogram, including older age, male, hyperlipidemia, smoking, high consumption of red meat, high consumption of salt, high consumption of dietary fiber, Helicobacter pylori infection, non-alcoholic fatty liver disease and chronic diarrhea. The model showed favorable discrimination values, with the area under the curve of the discovery and validation cohorts 0.775 (95% confidence interval (CI), 0.755-0.794) and 0.776 (95% CI, 0.744-0.807) respectively. The model was also well-calibrated, with Hosmer-Lemeshow test* P* = 0.370. In addition, the decision curve analysis revealed that the model had a higher net profit compared with either the screen-all scheme or the screen-none scheme.

**Conclusion:** In this prospective study, we established and validated a prediction model that incorporated a list of high-risk features related to colorectal ademomatous polyps occurrence, showing favorable discrimination and calibration values.

## Introduction

Colorectal cancer (CRC) has become the fifth most common cause of cancer-related death in China. About 555,477 individuals were diagnosed with CRC, and 286,162 patients died of CRC in 2020 1. CRC commonly develops from precursor lesions termed polyps, described as lumen that grow into a cavity. Colorectal ademomatous polyps have varying degrees of size and dysplasia 2. They may be categorized as pedunculated or sessile based on their gross morphology. According to the histological presentation, they can also be classified as neoplastic or non-neoplastic. Non-neoplastic polyps have no malignant potential and can be further subdivided into hyperplastic, hamartomatous and inflammatory polyps. Neoplastic polyps are adenomatous and serrated, with potential malignancy, representing a stage of CRC development. Tubular, tubular villous, and villous adenomas with varying degrees of villous characteristics are the three types of adenomas. Serrated polyps also include three distinct sub-categories: hyperplastic polyps, sessile serrated adenomas/polyps (SSA/P) and traditional serrated adenomas (TSA) 3.

Individuals with colorectal ademomatous polyps are easily diagnosed now by using the wider application of fecal occult blood testing, fecal immunohistochemical test, flexible sigmoidoscopy, colonoscopy, computed tomographic colonography or colon capsule endoscopy, etc. 4. Although colonoscopy is considered to be relatively safe and the gold standard for the diagnosis of colorectal ademomatous polyps, it still requires high-level experts due to the complicated operation and time-consuming. Meanwhile, patients may face tremendous burdens, including bowel preparation, time away from work, discomfort and financial considerations 5. In addition, colonoscopy is not well accepted among the general public. Therefore, screening for population-based polyps, especially using colonoscopy as the primary modality, remains a major challenge 6. For these reasons, identifying putative high-risk factors for the occurrence of colorectal ademomatous polyps may be more clinically beneficial and provide more insights for cancer prevention. The role of various modifiable lifestyle factors and associated comorbidities in polyps pathology has been documented and verified, especially in colorectal neoplasm 7. In terms of lifestyle factors, there are various known factors, including smoking, alcohol consumption, red meat, physical activities and obesity. As for comorbidities, there are chronic gastritis, inflammatory bowel disease (IBD), hyperlipemia, diabetes and hypertension 8.

Nomogram has been accepted as a reliable prediction model to quantify the risk of a clinical event by constructing a simple and intuitive graph 9. Therefore, developing a model based on high predictive parameters is critical to improve the detection rate in high-risk groups likely to develop CRC. This study aimed to identify a group of high-risk factors, construct a prediction model for the occurrence of colorectal ademomatous polyps, and avoid unnecessary surveillance and waste of medical resources.

## Methods

### Study population

**Figure 1:**
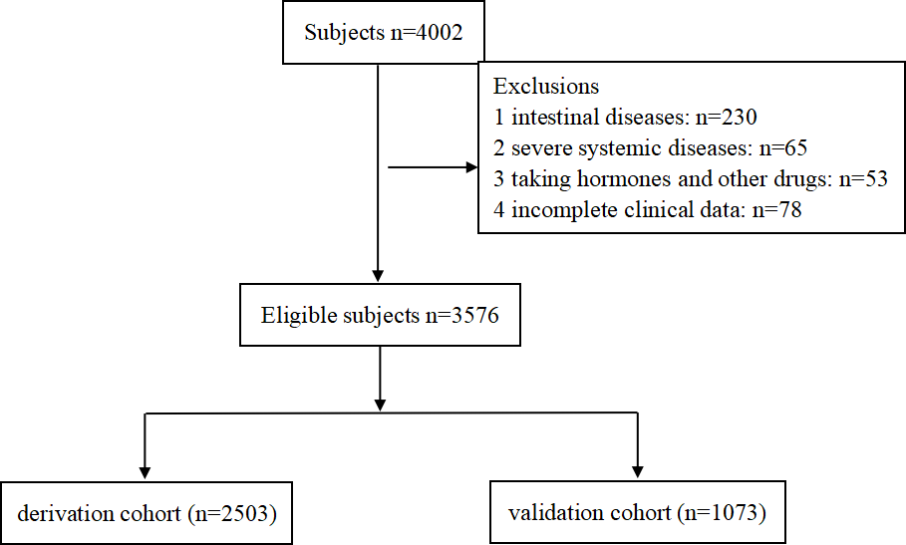
Flow diagram of the model's discovery and validation cohort.

A total of 3576 confirmed eligible participants, including 2520 colorectal ademomatous polyps and 1056 non-polyp controls, were enrolled in this prospective study from the Department of Gastroenterology in the First Affiliated Hospital of Nanjing Medical University from June 2019 to December 2021. For analysis purposes, we randomly divided all 3576 participants into a discovery cohort (2503, 70%) and a validation cohort (1073, 30%) (Fig. 1). Inclusion criteria included: (1) participants' age over 18 years old; (2) polyp cases with any colorectal ademomatous polyps detected under their first-time colonoscopy presently and confirmed by the postoperative tissue pathology; (3) eligible none-polyp controls without any history of colorectal polyps and verified by the colonoscopy in recent one year; (4) complete medical records; (5) participants who are willing to cooperate with the questionnaire survey. Exclusion criteria included: (1) the history of intestinal diseases: IBD, intestinal tuberculosis, familial adenomatous polyposis, P-J syndrome and intestinal lymphoma, etc.; (2) the history of severe systemic diseases: liver cirrhosis, metabolic syndrome, chronic kidney disease, malignant tumor, etc.; (3) resent use of lipid-lowering drugs and hormone or immunosuppressive agents; (4) incomplete clinical information or unwillingness to cooperate with the questionnaire survey. All colonoscopies were performed by board-certified gastroenterologists with over 2000 procedures. This study was approved by the Ethics Committee of the First Affiliated Hospital of Nanjing Medical University (2019-SR-020). All patients provided written informed consent, including data collection and analysis.

### Collection of demographic and clinical data

Demographic and clinical data for cases and controls were obtained from detailed interviews and electronic medical records according to structured questionnaire survey. The questionnaire content mainly focused on demographic data, anthropometric measurement, family history, comorbidity history and lifestyle factors.

Demographic data: age (18-45, 45-69, >69 years old), sex (male, female). Anthropometric measurements: body mass index (BMI), calculated by height and weight. Family history: the first-class family history of colorectal tumors. Comorbidity history: Helicobacter pylori (H. pylori) infection, non-alcoholic fatty liver disease (NAFLD), gallbladder diseases (gallbladder polyps or gallstones), diabetes mellitus, hypertension, hyperlipidemia (triglycerides above 1.7 mmol/L or total cholesterol above 5.7 mmol/L or low-density lipoprotein cholesterol above 3.4 mmol/L in the venous blood test, according to the 2019 Chinese Guideline for the Management of Dyslipidemia in Adults). Laboratory examinations: fasting blood glucose, blood routine examination (white blood cell, neutrophils, lymphocyte, monocyte, eosinophils, basophils, platelet). Chronic constipation: defecate less than 3 times per week and last for over 6 months, with hard and less stools. Chronic diarrhea: defecate over 3 times per week and last for over 6 months, defecate more than 200 grams a day, with undigested food, pus, blood or mucus. And lifestyle factors: smoking (current: one pack of cigarettes or more a week , last for over 1 year; former: over 5 years and have quitted; never), alcohol use (current: once or more a week, last for 1 year or more; former: over 5 years and have quited; never), high consumption of red meat (HCRM) (pork, beef, mutton, etc. 3 or more times a week), high consumption of greasy food (HCG) (2 or more times a week), high consumption of salt (HCS) (2 or more times a week), high consumption of pungency (HCP) (2 or more times a week), high consumption of dietary fiber (HCDF) (fruit or vegetables, every day), physical activity (manual worker; regular exercise: less than 1 hour per day, 5 or more times a week; more than 1 hour per day, 2 or more times a week; long-distance runners, once or more a week). These factors were chosen because of their hypothetical roles in the development of colorectal adenomatous polyps.

### Statistical analysis

Baseline characteristics of patients with and without colorectal ademomatous polyps in the discovery and validation cohorts were compared. Categorical variables were presented as the number (%) and assessed using the χ^2^ tests or Fisher's exact test appropriately. Continuous variables were described as median ± standard deviation (SD) and compared using Student's t-test. We used SPSS 24.0 and R software version 3.6.1 (R Foundation for Statistical Computing, Vienna, Austria) for statistical analysis. A two-sided *P*-value of < 0.05 was considered statistically significant.

### Identification of Independent Predictive Factors

In the discovery cohort, to address the impacts of over-fitting, the Least Absolute Shrinkage and Selection Operator (LASSO) regression method was applied using the glmnet package in R project 10, which is superior to univariate analysis. To select the optimal lambda (λ) parameters and corresponding coefficients, we performed 10-fold cross-validation with AUC maximum criteria. The λ via 1-SE (standard error) criteria was selected to screen for the best factors. Then we applied the multivariate logistic regression analysis to identify independent predictive factors of colorectal ademomatous polyps.

### Construction of the Nomogram

Based on the above results, a prediction model for colorectal ademomatous polyps was constructed using the rms package of R project, providing a visual tool for clinical application. The prediction model was represented by a nomogram based on independent risk factors identified by multivariate analysis. Briefly, the nomogram found the position of each variable on the corresponding axis, and found a point for each variable on the top rule; then all scores were added together and the total was collected. Finally, the corresponding risk probability of the individual colorectal ademomatous polyps was predicted with the lowest rule by adding the scores of all selected variables.

### Validation of the Nomogram

To verify the predictive ability of the model, the receiver operating characteristic (ROC) analysis, calibration curve and decision curve analysis (DCA) were used in both the discovery and validation cohorts. Discriminant ability means the ability of the nomogram to distinguish events from non-events by calculating the area under the curve (AUC) and the associated 95% confidence interval (CI). The range of AUC was 0.5-1.0, with 0.5 indicating random prediction and 1.0 perfect prediction. It is accepted that an AUC between 0.5 and 0.7 indicates low prediction accuracy, between 0.7 and 0.9 indicates moderate prediction accuracy, and above 0.9 indicates high prediction accuracy. Subsequently, the consistency between the predicted results and the actual results was evaluated by Hosmer-Lemeshow (H-L) test and calibration curve. The clinical utility of the model was assessed by DCA. The x-axis represented the percentage of threshold probability and the y-axis represented the net benefit of the predictive model. The net benefit was calculated by subtracting the proportion of false positives from the proportion of true positives and weighted by the relative harm of foregoing detection compared with the negative consequences of an unnecessary detection 11.

## Results

### Baseline characteristics of study participants

Among the 3576 study participants, 2520 (70%) were colorectal ademomatous polyps. Participants in the colorectal ademomatous polyps group were older, had higher BMI, were more likely to be male, smokers, high consumption of red meat, pungency, greasy food and salt, hyperlipidemia, NAFLD, gallbladder polyps, chronic diarrhea, hypertension and H. pylori infection, while had less proportion of high consumption of dietary fiber. Alcohol user, physical activity, family history of colorectal tumors, gallstones, constipation and blood routine examination were not significantly different between the groups. The validation cohort has similar characteristics to the discovery cohort. Details were shown in Table 1.

### Identification of Independent Predictive Factors

**Figure 2:**
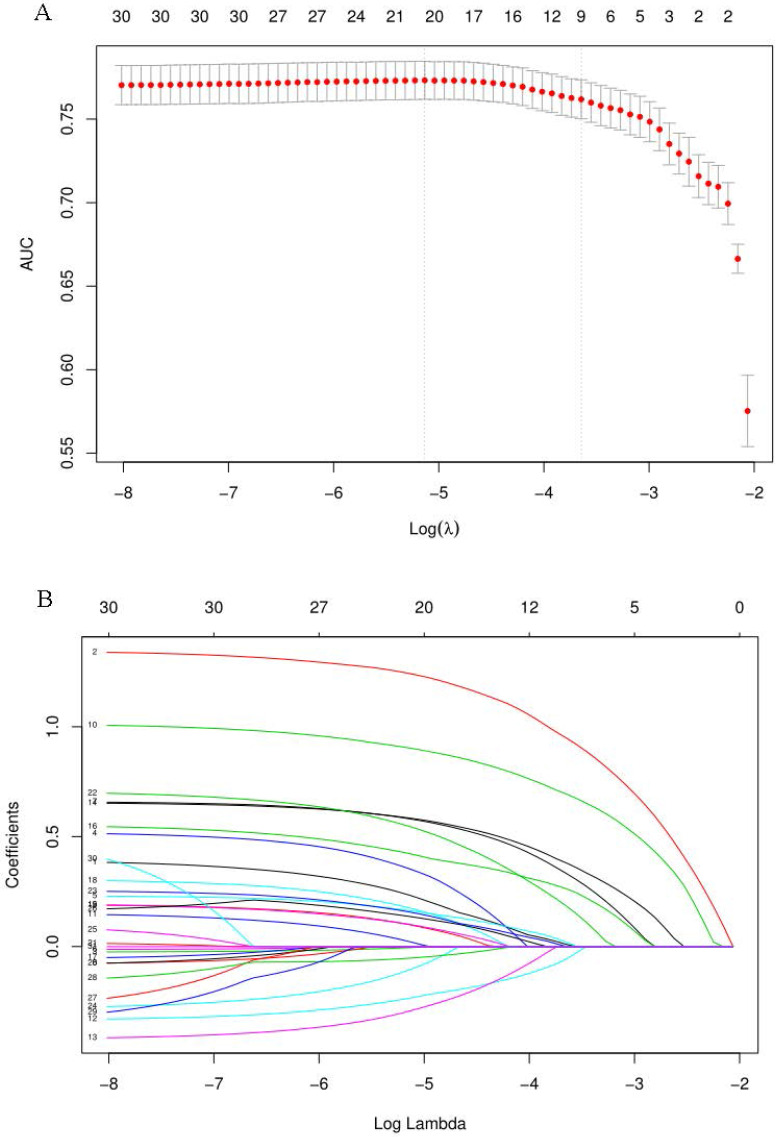
** Predictor selection using the LASSO regression analysis with 10-fold cross-validation. (A)** Tuning parameter (λ) selection of deviance in the LASSO regression based on the minimum criteria (left dotted line) and the 1-SE criteria (right dotted line). **(B)** A coefficient profile plot was created against the log (λ) sequence. In the present study, predictor's selection was according to the 1-SE criteria (right dotted line), where 10 non-zero coefficients were selected. LASSO: least absolute shrinkage and selection operator; SE: standard error.

When the AUC reached its maximum value, the most appropriate tuning parameter λ was 0.007, and the λ corresponding to 1-SE was 0.026 (Fig. 2A). 10 variables with non-zero coefficients were retained in the LASSO analysis (Fig. 2B). These variables included older age, male, hyperlipidemia, smoking, HCRM, HCS, HCDF, H. pylori infection, NAFLD and chronic diarrhea. To establish a predictive model for colorectal adenomatous polyps, we performed a multivariate logistic regression analysis based on the above variables selected by the LASSO regression model. Table 2 showed that HCDF was found to be independently correlated with a reduced risk of ademoma polyps. In addition, an elevated risk of ademoma polyps was independently observed in the older age, male, hyperlipidemia, smoking, HCRM, HCS, HCDF, H. pylori infection, NAFLD and chronic diarrhea.

### Construction of the nomogram

**Figure 3:**
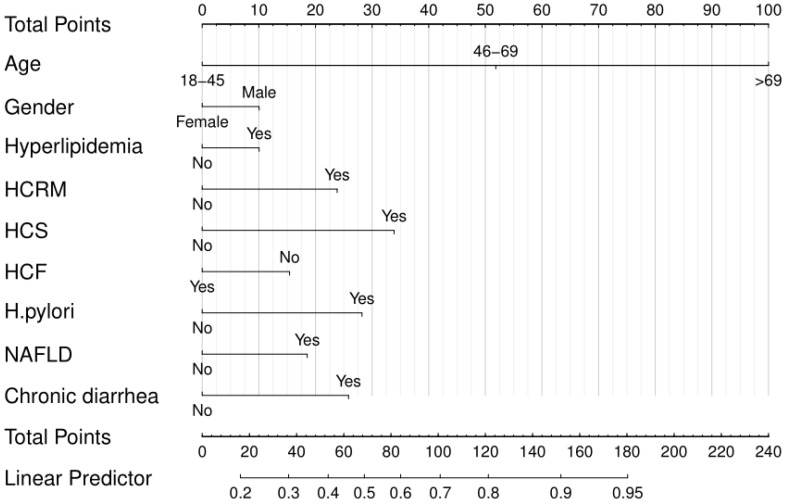
Nomogram for predicting colorectal ademomatous polyps risk and its algorithm.

To visualize the predictive model, a nomogram was constructed based on multivariate logistic regression (Fig. 3), including 10 significant predictors, thus providing a convenient, personalized tool to predict the probability of colorectal ademomatous polyps.

### Validation of the nomogram

**Figure 4:**
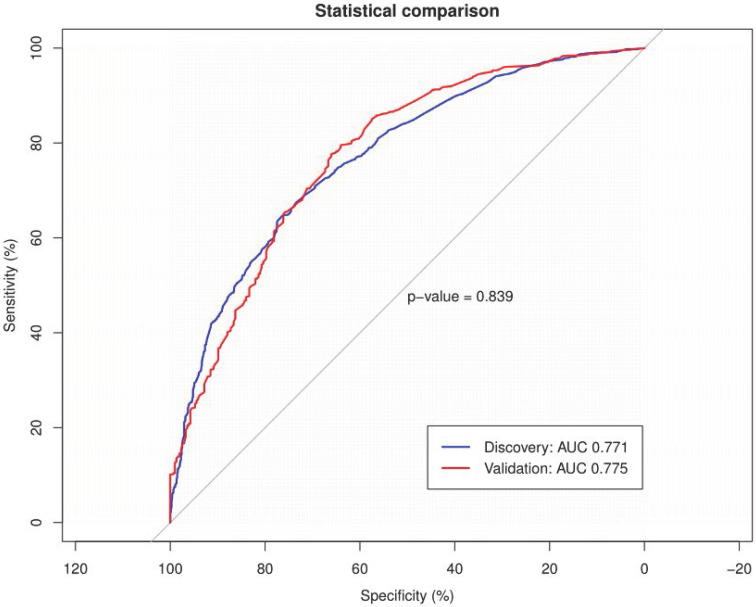
** The AUC of the discovery and validation cohorts were 0.775 and 0.776 respectively.** The blue line represented the ROC curve of the discovery cohort and the red line represented the ROC curve of the validation cohort. ROC: receiver operating characteristic; AUC: area under the curve.

**Figure 5:**
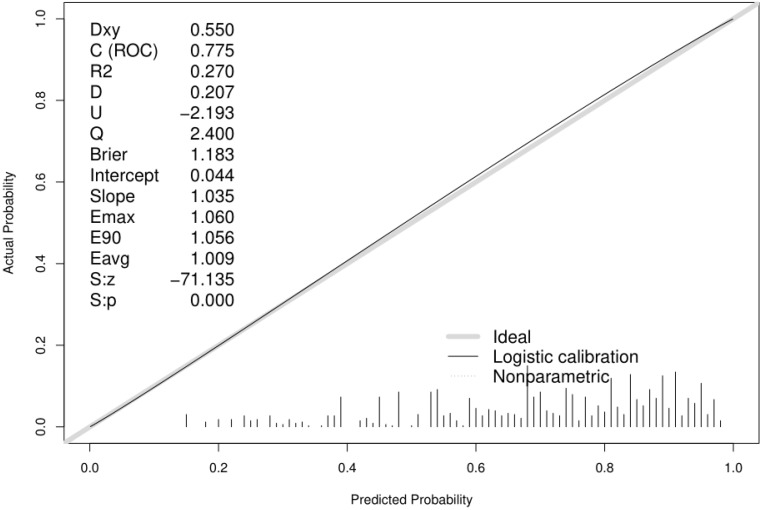
Calibration curve of the predictive model showing consistency between the predicted probability and observed probability (the H-L test, *P*=0.370, suggesting that it is of goodness-of-fit). The gray solid line represented a perfect prediction by an ideal model, and the black solid line shows the performance of the model.

**Figure 6:**
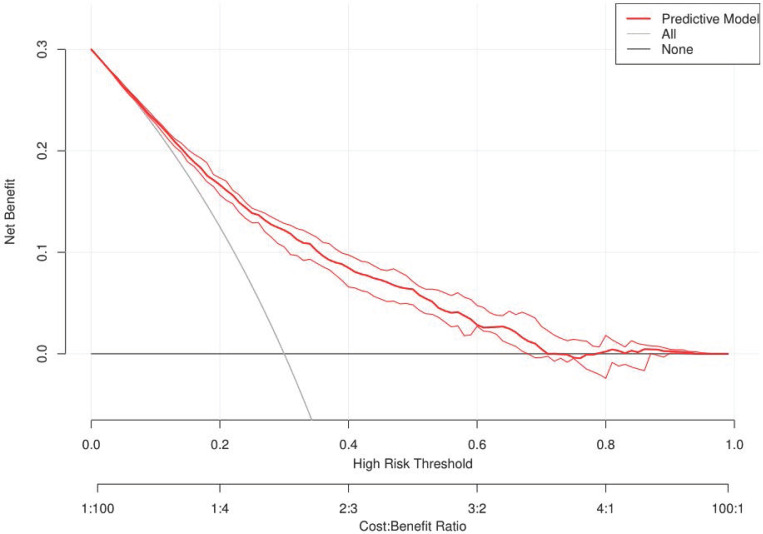
** DCA of the nomogram.** The red solid line represented the predictive model. The blue solid line represented the screen-all scheme. The black solid line represented the screen-none scheme. DCA: decision curve analysis.

To validate the performance of the resulting nomogram, we performed internal validation using an independent validation cohort. In Fig. 4, the nomogram was well distinguished, as shown, the AUC of the discovery cohort and the validation cohort were 0.775 (95% CI, 0.755-0.794 and 0.776 (95% CI, 0.744-0.807), respectively, with better predictive efficiency compared to other models (*p* < 0.05). Additionally, the proposed model was well-calibrated using the H-L test, yielding a non-significant *P* value of 0.370. As shown in Fig. 5, the calibration curve of the nomogram was very close to the 45-degree line, indicating that the nomogram exhibited favorable concordance between actual outcomes and predicted probabilities. The DCA curve (Fig. 6) showed that the nomogram model had a higher net profit in almost the entire threshold probability range compared with either the screen-all scheme or the screen-none scheme.

## Discussion

The impact of various modifiable lifestyle patterns and clinical risk factors on colorectal ademomatous polyps has been extensively studied 12. Our study revealed that older age, male, smoking, high consumption of red meat and salt, hyperlipidemia, H. pylori infection, NAFLD, and chronic diarrhea were all associated with an increased risk of colorectal ademoma polyps. A certain amount of dietary fiber was found to prevent colorectal adenomatous polyps, which had rarely been documented before. Therefore, we have established and validated a nomogram containing the above available variables for predicting the risk of developing colorectal adenomatous polyps, which could provide a visual tool for clinical use.

Gender and age are unmodifiable and important predictors for colorectal ademomatous polyps. Pooled studies have reported that the prevalence of colorectal adenomatous polyps increases progressively with age in both men and women and are more common in men than in women in every age group 13. There is now growing interest in studying dietary patterns and their association with colorectal ademomatous polyps 14. A healthy diet is characterized by high consumption of fruit and vegetables, while an unhealthy diet is characterized by high consumption of red meat, salt, sugar, and refined grains 15. Our study confirmed the previous research that red meat has an impact on the occurrence of colorectal ademomatous polyps. A meta-analysis of observational studies showed a 22% increased relative risk of adenomatous polyps in individuals with high versus low red meat intake, similar to serrated polyps. Aune et al. 16 found that a high intake of fresh vegetables and fruit was associated with a reduced risk of CRC. The reason may be that increasing the intake of fiber food would reduce the intestinal transit time and the exposure time of carcinogens, thereby reducing the risk of colorectal polyps. Our results showed that the risk of colorectal adenomatous polyps can be reduced with increased fruit and vegetable intake, which is consistent with previous studies. Therefore, moderate intake of red meat and salt, and increased intake of fiber food are recommended to reduce the risk of adenomatous polyps developing into neoplasia, including CRC.

About 50% of the global population is infected with H. pylori, and several researchers have found that H. pylori infection is associated with the occurrence of colorectal polyps 17, which is consistent with our results. Gastric H. pylori infection induces colorectal tumors by regulating the expression of serum gastrin, and then hypergastrinemia accelerates the proliferation of gastrointestinal mucosal cells. Chronic inflammation also generates DNA damage and enhances inflammation- related colon tumorigenesis 18. Therefore, for patients with H. pylori infection, we recommend early screening of colonoscopy to improve the early diagnosis rate of colorectal ademomatous polyps.

An association between NAFLD and colorectal ademomatous polyps was also observed in this study, consistent with previous studies, indicating a moderate association 19. The pathophysiological mechanism between fatty liver and adenomatous polyps is unclear. The potential hypotheses are based on insulin resistance and obesity-related inflammation, which promote cell proliferation, angiogenesis, and adiponectin expression. We also recommend that men over 45 years of age with fatty liver have a colonoscopy earlier than the normal population.

Our study found that chronic diarrhea was an independent risk factor for colorectal ademomatous polyps. However, few studies have been conducted on the association between intestinal dysfunction and colorectal adenomatous polyps, the molecular mechanism remain poorly understood. It may be related to active intestinal peristalsis, which may lead to intestinal epithelial cell proliferation, mucosal inflammation and intestinal flora imbalance 20.

Smoking is a significant and well-documented modifiable risk factor for colorectal adenomatous polyps. Studies have consistently shown that the proportion of colorectal adenomas in smokers is significantly higher than that in non-smokers 21. Previous studies have revealed that smoking status, duration and intensity were associated with an increased risk of colorectal polyps, which was consistent with our findings 22. Tobacco exposes smokers to many carcinogens that are thought to cause irreversible gene mutations in colorectal mucosa, leading to the formation of colorectal polyps 23. Some studies have revealed that hyperlipidemia promotes the formation of colorectal ademomatous polyps 24, which was in the same as our study. The specific mechanisms remain unclear, which may be connected to the release of inflammatory cytokines and an increase of insulin resistance 25.

The nomogram we constructed showed better discriminatory ability in the discovery and validation cohorts. Compared with the previously published Western colorectal adenomas detection model, the current model has more risk factors than the previous models, including various dietary factors. Shaukat et al 26 reported a simple score that taking into account age, male, BMI, family history of at least one first-degree relative with CRC, and smoking history for predicting the risk of advanced adenoma with general discrimination(AUC=0.64), but lack of validation. Wong et al 27 developed a new scoring system consisting of age, gender, BMI, family history, smoking and self-reported diabetes to estimate the possibility of colorectal neoplasia, with the c-statistic 0.62 for the discovery set and the validation set, respectively, indicating moderate discrimination.

Specific strengths and limitations deserve careful attention when interpreting our results. A major strength of our study is that most of variables included in this model are usually available from the patients' history, which ensures that these factors are readily available in clinical practice. The ROC curve results of our model showed that its sensitivity and specificity were very good, the calibration curves showed that the predicted probability was in good agreement with the actual probability, and the DCA also showed that the model had high clinical practical value. This study still has some limitations, which should be recognized and considered. First, these limit the applicability and generalizability of the nomogram due to the relatively small sample size of the validation cohort in our analysis. Moreover, since the lifestyle data may have subjective elements, the inherent recall bias is more or less unavoidable. Furthermore, some clinical features, such as the use of insulin, C-peptide, NSAIDs, and aspirin, are also important in the evaluation of polyps 28. Unfortunately, these variables were not available in our analysis, which may discount the power of our nomogram. However, we can continuously adjust the parameters in practical applications to make the results of the nomogram analysis more reliable. Finally, we were not able to obtain the histopathology report of each polyp to further evaluate different types of risk factors. Despite these limitations, our findings will provide important insights for designing effective colorectal polyps screening strategies in the future.

In conclusion, in this study, we developed and validated a model based on the most readily available clinical features for personalized prediction of colorectal adenomatous polyps. The model showed good calibration and discrimination values and clinical applicability, which is valuable for identifying asymptomatic individuals with coloretcal adenomatous polyps and selecting high-risk target groups for colonoscopy screening. We believe this model will be a good clinical decision-making support tool. However, larger prospective studies and external validations are necessary to confirm our findings and further optimize the model.

## Figures and Tables

**Table 1:** Demographic and clinical data of study participants

Variable	Discovery		Validation	
	Polyps (n=1753)	Non-Polyp (n=750)	Polyps (n=767)	Non-Polyp (n=306)
**Age, years**	58.5±11.4	49.8±13.6*	58.4±11.3	50.4±14.2*
**Gender, n (%)**				
Male, n (%)	1061(60.5)	393(52.4)*	480(62.6)	151(49.3)*
Female, n (%)	692 (39.5)	357(47.6)*	287(37.4)	155(50.7)*
**BMI, kg/m^2^**	23.9±3.1	23.5±3.5*	23.9±3.0	23.3±3.3*
**Smoking, n (%)**				
Never	1243(70.9)	605(80.7)*	240(68.7)	61(80.1)*
Former	276(15.7)	91(12.1)*	240(14.2)	61(11.4)*
Current	234 (13.3)	54(7.2)*	240(17.1)	61(8.5)*
**Alcohol user, n (%)**				
Never	1355(77.3)	596(79.5)	588(76.7)	234(76.5)
Former or Current	398(22.7)	154(20.5)	179(23.3)	72(23.5)
**HCRM, n (%)**				
No	795(45.4)	493(65.7)*	358(46.7)	193(63.1)*
Yes	958(54.6)	257(34.3)*	409(53.3)	113(36.9)*
**HCP, n (%)**				
No	1198(68.3)	545(72.7)*	517(67.4)	226(73.9)*
Yes	555(31.7)	205(27.3)*	250(32.6)	80(26.1)*
**HCG, n (%)**				
No	999(57.0)	540(72.0)*	425(55.4)	215(70.3)*
Yes	754(43.0)	210(28.0)*	342(44.6)	91(29.7)*
**HCS, n (%)**				
No	858(48.9)	557(74.3)*	390(50.8)	228(74.5)*
Yes	895(51.1)	193(25.7)*	377(49.2)	78(25.5)*
**HCDF, n (%)**				
No	468(26.7)	172(22.9))*	193(25.2)	67(21.9)
Yes	1285(73.3)	578(77.1)*	574(74.8)	239(78.1)
**Physical activity, n (%)**				
No	894(51.0)	398(53.1)	387(50.5)	149(48.7)
Yes	859(49.0)	352(46.9)	380(49.5)	157(51.3)
**Hyperlipidemia, n (%)**				
No	962(54.9)	495(66.0)*	408(53.2)	202(66.0)*
Yes	791(45.1)	255(34.0)*	359(46.8)	104(34.0)*
**FHCT, n (%)**				
No	1494(85.2)	659(87.9)	645(84.1)	267(87.3)
Yes	259(14.8)	91(12.1)	122(15.9)	39(12.7)
**NAFLD, n (%)**				
No	1169(66.7)	612(81.6)*	513(66.9)	263(85.9)*
Yes	584(33.3)	138(18.4)*	254(33.1)	43(14.1)*
**Gallstone, n (%)**				
No	1536(87.6)	677(90.3)	680(88.7)	284(92.8)
Yes	217(12.4)	73(9.7)	87(11.3)	22(7.2)
**Gallbladder polyps, n (%)**				
No	1613(92.0)	712(94.9)*	705(91.9)	296(96.7)*
Yes	140(8.0)	38(5.1)*	62(8.1)	10(3.3)*
**Constipation, n (%)**				
No	1550(88.4)	666(88.8)	679(88.5)	274(89.5)
Yes	203(11.6)	84(11.2)	88(11.5)	32(10.5)
**Chronic diarrhea, n (%)**				
No	1501(85.6)	702(93.6)*	668(87.1)	292(95.4)*
Yes	252(14.4)	48(6.4)*	99(12.9)	14(4.6)*
**Hypertension, n (%)**				
No	1196(68.2)	595(79.3)*	482(62.8)	239(78.1)*
Yes	557(31.8)	155(20.7)*	285(37.2)	67(21.9)*
**Diabetes mellitus, n (%)**				
No	1576(89.9)	697(92.9)*	698(91.0)	279(91.2)
Yes	177(10.1)	53(7.1)*	69(9.0)	27(8.8)
**H.pylori, n (%)**				
No	1168(66.6)	602(80.3)*	503(65.6)	247(80.7)*
Yes	585(33.4)	148(19.7)*	264(34.4)	59(19.3)*
**WBC, ×10^9^ /L**	5.7±1.6	5.7±1.7	5.8±1.9	5.7±1.8
**Neutrophils, ×10^9^ /L**	3.3±1.2	3.4±1.5*	3.4±1.6	3.4±1.6
**Lymphocyte, ×10^9^ /L**	1.8±0.6	1.7±0.6*	1.8±0.6	1.7±0.6
**Monocyte, ×10^9^ /L**	0.4±0.1	0.4±0.2	0.4±0.1	0.4±0.2
**Eosinophils, ×10^9^ /L**	0.1±0.1	0.1±0.1	0.1±0.1	0.1±0.1
**Basophils, ×10^8^ /L**	0.3±0.2	0.3±0.2	0.3±0.2	0.3±0.2
**Platelet, ×10^9^ /L**	197.4±55.8	203.4±57.6*	199.4±59.9	204.8±66.8
**Serum glucose, ×10^9^ /L**	5.0±1.1	4.9±1.2*	4.9±1.1	5.1±1.6*

Continuous variables are expressed as mean ± SD and categorical variables are expressed as number (%). Abbreviations: BMI: body mass index; HCRM: high consumption of red meat; HCP: high consumption of pungency; HCG: high consumption of greasy; HCS: high consumption of salt; HCDF: high consumption of dietary fiber; FHCT: family history of colorectal tumors; NAFLD: non-alcoholic fatty liver disease; H.pylori: helicobacter pylori; WBC: white blood cell. *A two-tailed significant difference P<0.05 between patients with and without ademomatous polyps.

**Table 2:** Univariate and multivariate logistic regression analysis in the discovery cohort

Variables	Univariate		Multivariate	
	OR (95%CI)	*P* value	OR (95%CI)	*P* value
**Age, years**		<0.001		<0.001
18-45	Ref.		Ref.	
46-69	3.22 (2.61-3.97)	<0.001	3.97 (3.13-5.03)	<0.001
>69	9.93 (6.70-14.72)	<0.001	14.20 (9.33-21.5)9	<0.001
Male, %	1.53 (1.29-1.82)	<0.001	1.27 (1.02-1.58)	0.031
**Smoking, %**		<0.001		0.032
Never	Reference		Reference	
Former	1.48 (1.14-1.91)	0.003	1.18 (0.87-1.61)	0.288
Current	2.11 (1.55-2.88)	<0.001	1.60 (1.12-2.30)	0.010
Hyperlipidemia, %	1.60 (1.34-1.91)	<0.001	1.24 (1.02-1.52)	0.035
HCRM, %	2.31 (1.94-2.76)	<0.001	1.83 (1.48-2.25)	<0.001
HCS, %	3.01 (2.49-3.64)	<0.001	2.55 (2.05-3.16)	<0.001
HCDF, %	0.82 (0.67-0.99)	0.048	0.69 (0.55-0.86)	0.001
H.pylori, %	2.04 (1.66-2.50)	<0.001	1.98 (1.58-2.48)	<0.001
NAFLD, %	2.22 (1.80-2.73)	<0.001	1.55 (1.23-1.96)	<0.001
Chronic diarrhea, %	2.46 (1.78-3.39)	0.001	1.87 (1.32-2.66)	<0.001

Abbreviation: OR: odds ratio; CI: 95% confidence interval; HCRM: high consumption of red meat; HCS: high consumption of salt; HCDF: high consumption of dietary fiber; H.pylori: helicobacter pylori; NAFLD: non-alcoholic fatty liver disease.

## Abbreviations

CRC: colorectal cancer
SSA/P: sessile serrated adenomas/polyps
TSA: traditional serrated adenomas
IBD: inflammatory bowel disease
BMI: body mass index
HCRM: high consumption of red meat
HCP: high consumption of pungency
HCG: high consumption of greasy
HCS: high consumption of salt
HCDF: high consumption of dietary fiber
FHCT: family history of colorectal tumors
NAFLD: non-alcoholic fatty liver disease
H. pylori: helicobacter pylori
WBC: white blood cell
